# Adherence to the Mediterranean Diet and Incidence of Pre-Frailty and Frailty in Community-Dwelling Adults 70+: The 3-Year DO-HEALTH Study

**DOI:** 10.3390/nu14194145

**Published:** 2022-10-06

**Authors:** Stephanie Gängler, Hanna Steiner, Michael Gagesch, Sophie Guyonnet, E. John Orav, Arnold von Eckardstein, Walter C. Willett, Heike A. Bischoff-Ferrari

**Affiliations:** 1 Center on Aging and Mobility, University Hospital Zurich, Zurich City Hospital-Waid and University of Zurich, 8006 Zurich, Switzerland; 2 Department of Aging Medicine and Aging Research, University Hospital Zurich and University of Zurich, 8006 Zurich, Switzerland; 3 University Clinic for Aging Medicine, Zurich City Hospital-Waid, 8037 Zurich, Switzerland; 4 Gérontopôle de Toulouse, Institut du Vieillissement, Centre Hospitalo-Universitaire de Toulouse, 31059 Toulouse, France; 5 UMR INSERM 1027, University of Toulouse III, 31059 Toulouse, France; 6 Department of Biostatistics, Harvard T.H. Chan School of Public Health, Boston, MA 02115, USA; 7 Institute for Clinical Chemistry, University Hospital Zurich, 8091 Zurich, Switzerland; 8 Department of Nutrition, Harvard T.H. Chan School of Public Health, Boston, MA 02115, USA; 9 Department of Epidemiology, Harvard T.H. Chan School of Public Health, Boston, MA 02115, USA

**Keywords:** dietary patterns, inflammaging, aging, frailty, Mediterranean diet

## Abstract

The Mediterranean diet has been associated with many health benefits. Therefore, we investigated whether the degree of adherence to the Mediterranean diet at baseline, or changes in adherence over time, were associated with the incidence of pre-frailty or frailty in generally healthy older adults. This study used the DO-HEALTH trial data. We evaluated Mediterranean diet adherence with Panagiotakos’ MedDietScore at baseline and at 3-year follow-up; frailty was assessed annually with the Fried frailty phenotype. We used minimally and fully adjusted mixed logistic regression models to estimate the exposure–disease relationship. We included 1811 participants without frailty at baseline (mean age 74.7 years; 59.4% women). Baseline adherence, as reflected by the MedDietScore, was not associated with becoming pre-frail [OR(95%CI) = 0.93 (0.83–1.03) for five-point greater adherence] or frail [OR(95%CI) = 0.90 (0.73–1.12) for five points]. However, a five-point increase in the MedDietScore over three years was associated with lower odds of becoming pre-frail [OR(95%CI) = 0.77 (0.68–0.88)] and frail [OR(95%CI) = 0.77 (0.64–0.92)]. In generally healthy and active older adults, baseline adherence to the Mediterranean diet was not associated with the incidence of pre-frailty or frailty over a 3-year follow-up. However, improved adherence to the Mediterranean diet over time was associated with significantly lower odds of becoming pre-frail or frail.

## 1. Introduction

Frailty is a hallmark for accelerated aging [1], leading to an increased risk of falls, hospital admission and mortality [2]. The prevalence of frailty in unselected community-dwelling adults aged 65 and older has been reported to be 17% in ten European countries [3]. The at-risk state of pre-frailty has been observed to be twice as common in this age group (42.3%) compared to frailty, although figures vary by country [3]. Given the growing number of older adults in both the US and Europe [4], the prevention of frailty has become a public health priority [5]. 

So far, few studies have investigated the effects of dietary patterns on frailty. Instead of looking at individual foods or nutrients, pattern analysis has examined the effects of an overall diet and taken into account the synergies between the individual nutrients [6]. Several studies found that adherence to a Mediterranean dietary pattern is associated with decreased cardiovascular and all-cause mortality [7,8,9]. These benefits are explained by its healthy dietary components, including large amounts of fresh vegetables, fruits, olive oil and whole-grain products, as well as healthy protein sources including fish, pulses, nuts and, occasionally, poultry [10]. Additionally, it has been suggested that the Mediterranean diet reflects a lifestyle that extends to being physically active and enjoying social interactions with friends and family [10]. 

One of the suggested mechanisms for how the Mediterranean diet may influence health is the reduction of inflammation [11], and frailty has been associated with increased inflammation [12].

Conversely, a diet poor in antioxidant micronutrients (i.e., vitamin C, E, carotenoids, and polyphenols) and rich in saturated and trans-fatty acids prompts the immune system towards a pro-inflammatory state [11]. This results in enhanced production of pro-inflammatory cytokines, such as C-reactive protein (CRP) and interleukin (IL)-6 [11]. As a result, several organ systems may suffer, including muscle wasting [13], arteriosclerosis [14], and cognitive decline [15], increasing the risk of frailty [12].

In several [16,17,18,19,20,21,22,23,24], but not all [25,26,27,28] observational studies, the Mediterranean diet was inversely associated with frailty status. However, among these prior studies, only one used repeated dietary assessments [22]. Further, only a few studies [16,26,28] have investigated the association between the Mediterranean diet and pre-frailty, and all found no significant association.

Therefore, we investigated the association between the Mediterranean diet and both incident pre-frailty and frailty. We took advantage of the large DO-HEALTH trial data set of 2157 adults, aged 70 and older, followed for 3 years with prospective assessments of both frailty and diet. Additionally, we explored whether a possible association between adherence to the Mediterranean diet and frailty could be explained by changes in the inflammatory biomarkers IL-6 or hsCRP.

## 2. Methods

### 2.1. Study Design and Participants

This is a post-hoc analysis based on previously prospectively collected data from DO-HEALTH, a randomized, double-blind, placebo-controlled trial [29]. DO-HEALTH investigated the effects of vitamin D3, omega-3 fatty acids, and a home-based exercise program alone and in combination on healthy aging [30]. Participants were enrolled in seven study sites located in five countries (Switzerland, Austria, Germany, France, and Portugal). From 2012 to 2014, 2157 participants were enrolled, aged 70 years and older, living independently at home, with no major health event in the five years before enrollment, and who had sufficient mobility to come to the study centers, and good cognitive status (Mini-Mental State Examination (MMSE) score ≥24). The final follow-up was in November 2017, and median follow-up was 2.99 years [30]. Frailty was one of the predefined secondary endpoints of DO-HEALTH [29]. All participants provided written informed consent. The study and the present analyses were approved by the responsible ethics committee (BASEC Nr. 2021-00913).

### 2.2. Frailty Assessment

Pre-frailty and frailty were assessed at baseline and annually over three years of follow-up. We operationalized frailty according to the five domains of the Fried physical frailty phenotype [1], i.e., weakness, fatigue, involuntary weight loss, low gait speed and low activity level. A few adaptions to fit the variables available from the DO-HEALTH dataset were made. For weakness, grip-strength was recorded in kilopascal (kPa) from the best of three consecutive measurements with the dominant hand using a Martin Vigorimeter (KLS Martin Group, Tuttlingen, Germany). Cut-points were defined by the lowest quintile, stratified by sex and age group (<75 year and ≥75 years), as was performed by Fried et al. in their landmark study [31]. Fatigue was operationalized by a positive answer to the self-reported question: “In the last month, have you had too little energy to do things you wanted to?” from the Survey of Health, Aging and Retirement in Europe (SHARE) questionnaire [32]. Involuntary relevant weight loss was defined as >4.5 kg in one year or >5% in one year, as measured at a follow-up visit. Low gait speed was defined as ≤0.65 m/s (men ≤173 cm, women ≤159 cm), and ≤0.76 m/s (men >173 cm, women >159 cm) from the short physical performance battery [33]. Finally, low activity level was defined as an answer of “less than once a week” to the self-report question: “How often do you engage in activities that require a low or moderate level of energy such as gardening, cleaning the car, or doing a walk?” from the SHARE questionnaire [29]. We classified frailty by three categories: 0 points = robust, 1–2 points = pre-frail, 3–5 points = frail. Participants with a missing component of the frailty score, who could not be reliably classified into one of the three categories mentioned, were excluded from the analysis.

### 2.3. Inflammatory Biomarkers

DO-HEALTH collected fasting blood samples at baseline and at each yearly visit, processed them immediately and aliquoted them into 0.5 mL tubes for storage at −80°C. Every three months, the samples were shipped in dry-ice containers to the central DO-HEALTH biobank (Allschwil, Switzerland). The samples were stored at −80°C until analysis. IL-6 and hsCRP concentrations were analyzed at the Institute for Clinical Chemistry of the University Hospital Zurich, Switzerland (ISO Certified ISO/IEC 17025) under standardized procedures and with regular quality control. Biomarker concentrations were determined in plasma using heparin tubes.

CRP concentrations were measured using a latex-particle enhanced immunological turbidity assay on a Roche Cobas 8000 with a c701 module. The limit of detection was 0.3 mg/L with a calibrated range of 0.3–350 mg/L. The coefficients of variation for the low- and high-concentration quality control samples were 2.4% and 2.8%, respectively.

IL-6 concentrations were determined with electrochemiluminescence immunoassay (ECLIA) on a Roche Cobas c602. The limit of detection was 1.5 ng/L with a calibrated range of 1.5–5000 ng/L. The coefficients of variation for the low- and high-concentration quality control samples were 2.7% and 1.9%, respectively.

Values below the detection limit were imputed as half the respective detection limit.

### 2.4. Dietary Assessment

In DO-HEALTH, dietary assessments were performed at baseline and at year three using the 216-item DO-HEALTH food frequency questionnaire (FFQ) designed for older adults. The FFQ structure was based on food groups, containing foods frequently consumed across Europe [28].

To evaluate adherence to a Mediterranean dietary pattern, we calculated the Mediterranean diet score established by Panagiotakos et al. in 2007 (referred to as the MedDietScore) [8]. As the primary exposures for our analyses, we used both the baseline MedDietScore and the change in MedDietScore (ΔY3-BL) between baseline and year three. To calculate daily energy intake, we used a nutrition table specifically developed for DO-HEALTH that is applicable to different European countries.

### 2.5. Statistical Analysis

Population characteristics are displayed overall and by age group (70–74 and ≥75 years). Normally distributed variables are presented as mean ± standard deviation, and non-normal variables as median and interquartile range. Categorical variables are presented as frequency and percentage. For our main analyses assessing the association between the baseline MedDietScore and incident frailty (frailty score >2) or pre-frailty and frailty combined (i.e., frailty score ≥1, which we will refer to as “at least pre-frailty”), we used mixed-effects logistic regression models. Separate generalized estimating equations (GEE) models were run with frailty and with at least pre-frailty as the outcomes, with each participant appearing in the analysis three times (years 1, 2, and 3). For each outcome, two models were run with minimal and full adjustments for potential confounders based on the directed acyclic graphs generated in DAGitty [34]. Model 1 adjusted for sex, age, study site and the DO-HEALTH design variables, which include treatment (vitamin D3, omega-3 fatty acids, exercise), time, treatment and time interaction, fall in the year prior to DO-HEALTH recruitment and a spline at 85 years. Model 2 was additionally adjusted for BMI, daily energy intake, self-reported number of comorbidities, education years, MoCA score, and living status. Associations between change in the MedDietScore (ΔY3-BL) and incident frailty over time were examined using similar GEE models with additional covariate adjustment for baseline MedDietScore. An unstructured covariance structure was used for all models, and the results are expressed as odds ratios with their 95% confidence interval or beta-coefficients with standard error.

In exploratory analyses, we examined the association between the MedDietScore and changes from baseline in inflammatory markers (IL-6 and hsCRP). The changes in inflammatory markers from baseline after 1, 2, and 3 years of follow-up were used as repeated continuous outcomes in a linear-mixed effects model with random intercepts for participant. This model was additionally adjusted for the baseline inflammatory marker concentration.

The pre-specified subgroups of interest for the main outcomes (incident frailty and pre-frailty) include sex (men, women), body mass index (≥30 kg/m^2^ and <30 kg/m^2^), age group (≥75 and <75 years), physical activity (inactive = 0 times/week and active = 1–7 times/per week) and study site. We tested for significant interaction between the MedDietScore and the subgroup variables, and, if *p* < 0.05, we conducted the subgroup analysis. Statistical significance was evaluated at the 5% level. All analyses were done in SAS (v9.4 SAS Institute Inc., Cary, NC, USA) and R (v.4.0.3, R Core Team, Boston, MA, USA) in R Studio (v.1.3.1093, RStudio Team Boston, MA, USA).

## 3. Results

### 3.1. Sample Characteristics

Of the 2157 participants in the DO-HEALTH dataset, 346 were excluded because they had a missing element of the five components of the frailty score (n = 55), reported excessive calorie intake (≥5000 kcal/day in men and ≥4000 kcal/day in women; n = 78), were frail at baseline (n = 56) or had no follow up data (n = 157) (Figure S1). Of the included 1811 participants, 1076 (59.4%) were women, and the mean age was 74.74 years (SD 4.27). The adherence to the MedDiet and the daily energy intake did not differ between the two age groups. Older participants had higher IL-6 and CRP concentrations at baseline (Table 1). The characteristics of the study sample that was robust (frailty score = 0) at baseline can be found in Table S1 in the Supplement. Over the course of follow-up, the proportion of frail individuals increased from 2.5% to 3.8%, and the proportion of pre-frail individuals increased from 49.2% to 56.4%, at year 1 and year 3, respectively (Table S2).

### 3.2. Adherence to the MedDiet and Frailty

For the analysis based on MedDietScore at baseline, we did not find any significant association with the incidence of pre-frailty or frailty over three years of follow-up (Table 2). There were no significant interactions of the subgroup variables of interest and baseline MedDietScore (Table S3).

For the analysis based on the change in the MedDietScore (ΔY3-BL) from baseline to year three, a five-point increase in the MedDietScore was associated with lower odds of pre-frailty [OR (95%CI) = 0.77 (0.68–0.88), *p* < 0.001] and frailty [OR (95%CI) = 0.77 (0.64–0.92), *p* = 0.005], both in the minimally and fully adjusted models (Table 2).

There were no significant interactions of the subgroup variables of interest and the change in the MedDietScore (Table S3).

### 3.3. Adherence to the MedDiet and Inflammatory Biomarkers

For our mechanistic exploratory analysis, we analyzed the association between inflammation and MedDiet adherence.

The change in IL-6 concentrations from baseline was not associated with higher adherence to the MedDiet in either the minimally or fully adjusted models for neither the baseline MedDietScore nor the change in MedDietScore (ΔY3-BL) from baseline. In the minimally adjusted model, for a five-point higher baseline MedDietScore, the CRP concentration declined by 0.181 mg/L (SE 0.09, *p* = 0.046). This association was not significant in the fully adjusted model. There was no association between the change in CRP and the change in MedDietScore (ΔY3-BL) (Table 2).

## 4. Discussion

In this prospective study, baseline adherence to the MedDiet did not predict incident pre-frailty or frailty in the DO-HEALTH study sample of European community-dwelling adults aged 70+. However, a five-point improvement in the MedDietScore from baseline to the 3-year follow-up (ΔY3-BL) was associated with significantly reduced odds of both pre-frailty and frailty, independent of age and sex. In our exploratory mechanistic analysis, this apparent benefit could not be explained by a change in the inflammatory state represented by IL-6 or hsCRP.

Our finding that participants with a higher adherence to the MedDietScore at baseline did not have a lower odds of incident frailty is not in line with the findings of a recent meta-analysis by Kojima et al. [35] including 5789 older adults. In that analysis, a significant association between greater adherence to a Mediterranean diet and incident frailty was documented. Participants in the highest tertile of the Mediterranean diet score at baseline had a 56% reduction in the odds of incident frailty [OR (95%CI) = 0.44 (0.31–0.64, *p* < 0.001], compared to the lowest tertile. The absence of an association between baseline adherence to the MedDietScore and incident frailty may in part be explained by the generally healthy and active trial population enrolled in DO-HEALTH, with a very small number of incident frailty cases.

On the other hand, we did find a 23% reduction in the odds of becoming frail among those participants who improved their MedDietScore (ΔY3-BL) by five points over the three years of follow-up in DO-HEALTH. While the DO-HEALTH participation may have stimulated healthy eating habits and led to a change in diet and related risk of frailty, we cannot exclude the possibility that our findings are explained by reversed causality. The latter would suggest that participants experiencing frailty symptoms started to change their diet towards a lower MedDietScore. To the best of our knowledge, this is the first study to investigate the association of a change in the MedDietScore within an individual over time with frailty. Nonetheless, the magnitude of our effect size is consistent with a cross-sectional assessment among 1740 Greek adults aged 65 years and older, where each additional unit in the MedDietScore was associated with a 5% reduction in the odds of being frail [18]. Also in line with our findings, in 71,941 women aged 60 years and older from the Nurses’ Health Study, the relative risks of being frail per 1-SD increase in three different Mediterranean diet scores ranged from 0.87 to 0.93 [22].

Regarding our mechanistic exploration by a change in inflammatory state, we could not find any significant association between higher MedDiet adherence and changes in inflammatory biomarkers over time. As outlined in the introduction, our hypothesis was that the high amount of omega-3-fatty acids in the Mediterranean dietary pattern may reduce the risk of frailty through an anti-inflammatory benefit [11,36]. Alternative molecular pathways, not captured in our analyses, include a potential decrease of oxidative stress through high fruit and vegetable content [35].

## 5. Strengths and Limitations

To the best of our knowledge, this is the first study investigating the change in adherence to the MedDiet with regard to incident frailty. Further, the MedDietScore used in this analysis has a high validity compared to other available scores [37]. Moreover, the five-country multicentric design of DO-HEALTH supports generalizability to a generally healthy and active European population aged 70 years and older.

There are also limitations. While participants did not receive any advice or instructions related to diet, it is possible that participants improved their diet through the course of the trial, since the trial tested healthy lifestyle interventions, which may explain the documented association between improvement in the MedDietScore and the lower odds of incident frailty. In addition, we made the assumption that there was a gradual change in the MedDietScore from baseline to year 3 without having measured it. Further, the FFQ assessment of dietary intake carries the risk of recall bias [38]. Due to the observational nature of the study, residual confounding due to unmeasured factors may remain. Finally, DO-HEALTH does not reflect a population-based sample as participants were pre-selected as generally healthy older adults.

## 6. Conclusions

In this study, we found that increasing adherence to the MedDiet (ΔY3-BL) over time was associated with reduced odds of becoming pre-frail and frail. Nonetheless, our results need to be interpreted with caution, as baseline adherence alone was not associated with incident frailty. In fact, we cannot exclude the possibility that our findings about the change in adherence may be due to reverse causation: older adults changing their diet in response to frailty development. This study can be seen as hypothesis-generating and future interventional studies are warranted to confirm our findings.

## Figures and Tables

**Table 1:** Baseline characteristics of the study population without frailty at baseline.

Characteristics	Overall n = 1811	70–74 Years n = 1062	≥75 Years n = 749	*p*-Value
Age, mean (SD), years	74.74 (4.27)	71.88 (1.38)	78.79 (3.66)	<0.001
Women, No. (%)	1076 (59.4)	638 (60.1)	438 (58.5)	0.527
Education, mean (SD), years	12.92 (4.19)	13.27 (4.05)	12.42 (4.33)	<0.001
BMI, mean (SD) ^b^	26.15 (4.11)	26.11 (4.20)	26.21 (3.98)	0.626
MoCA score, median (IQR) ^c^, (0–30) points	26.00 (24.00, 28.00)	27.00 (24.00, 28.00)	26.00 (24.00, 28.00)	<0.001 ^a^
Sum of comorbidities, median (IQR)	1.00 (1.00, 2.00)	1.00 (1.00, 2.00)	2.00 (1.00, 3.00)	<0.001 ^a^
Pre-Frail, n (%)^d^	776 (42.8)	432 (40.7)	344 (45.9)	0.030
Physical activity volume, median (IQR), MET-h/week ^e^	27.39	30.00 (14.81, 54.00)	23.95 (10.00, 48.75)	<0.001 ^a^
Serum IL-6, median (IQR), ng/L	2.50 (1.50, 3.90)	2.20 (1.50, 3.40)	2.90 (1.90, 4.40)	<0.001 ^a^
Serum CRP, median (IQR), mg/L	1.50 (0.80, 2.80)	1.40 (0.80, 2.70)	1.60 (0.90, 3.00)	0.016 ^a^
Physical activity, N (%)				0.005
None	284 (15.7)	144 (13.6)	140 (18.7)	
1–2 times/week	554 (30.6)	320 (30.2)	234 (31.3)	
≥3 times/week	971 (53.7)	597 (56.3)	374 (50.0)	
Faller = Yes (%)	734 (40.5)	402 (37.9)	332 (44.3)	0.007
MedDietScore, mean (SD), (0–55) points	37.16 (4.90)	37.22 (4.76)	37.07 (5.10)	0.506
Energy intake (median (IQR)), kcal/day	2441 (1969, 2986)	2431 (1966, 2971)	2477 (1974, 3000)	0.445 ^a^

Abbreviations: IQR, interquartile range. ^a^ For these non-normal distributed variables, the Wilcoxon test was used. ^b^ Body mass index (BMI) was calculated as weight in kilograms divided by height in meters squared. Higher BMI values reflect overweight (≥25) and obesity (≥30). ^c^ The Montreal Cognitive Assessment (MoCA) is a screening test for mild cognitive dysfunction and has a range of 0 to 30 points, in which higher scores are better and scores greater than 26 suggest normal cognitive function. ^d^ Frailty was defined according to the five domains of the Fried physical frailty phenotype: exhaustion, weight loss, slowness, low activity and weakness. We have classified the results in the following way: 0 = robust, 1–2 = pre-frail, 3–5 = frail. ^e^ Weekly volume of physical activity was estimated based on the Nurses’ Health Study questionnaire on physical activity, in which energy expenditure of different activities in metabolic equivalent tasks (METs) of activities based on the Compendium of Physical Activities were summed over the previous week.

**Table 2:** Odds ratios for the incidence of frailty and at least pre-frailty by five points of MedDietScore.

	Model	Per 5-Point Higher MedDietScore at Baseline	Per 5-Point Change in the MedDietScore from Baseline to 3-Year Follow-Up (Δ_Y3-BL_) ^a^
		Odds Ratio (95%CI)	Odds Ratio (95%CI)
Incidence of at least pre-frailty	1	0.91 (0.82–1.00); *p* = 0.057	0.77 (0.68–0.88); *p* < 0.001
	2	0.93 (0.83–1.03); *p* = 0.17	0.77 (0.68–0.88); *p* < 0.001
Incidence of frailty	1	0.87 (0.71–1.06); *p* = 0.157	0.72 (0.61–0.86); *p* < 0.001
	2	0.90 (0.73–1.12); *p* = 0.336	0.77 (0.64–0.92); *p* = 0.005
		Beta-coefficient (Standard Error)	Beta-coefficient (Standard Error)
Change in IL-6 (ng/L)	1	0.002 (0.09); *p* = 0.984	−0.16 (0.11); *p* = 0.149
	2	0.012 (0.10); *p* = 0.900	−0.128 (0.12); *p* = 0.266
Change in CRP (mg/L)	1	−0.181 (0.09); *p* = 0.046	−0.096 (0.11); *p* = 0.377
	2	−0.157 (0.10); *p* = 0.092	0.017 (0.11); *p* = 0.882

Model 1: Adjusted for sex, age, study site and the DO-HEALTH design variables, which are treatment (vitamin D, omega-3s, exercise), time, treatment and time interaction, fall in previous year and spline at 85 years. Model 2: Adjusted additionally for BMI, number of comorbidities, education years, MoCA score, energy intake (kcal/day) and living status (alone, with spouse/relative). ^a^ Additionally adjusted for the baseline MedDietScore.

## Data Availability

In a first step, no data will be made available to researchers external to the DO-HEALTH Research Group to allow primary researchers to fully exploit the dataset. The data will be shared in a second step according to a controlled access system.

## Supplementary Materials

The following supporting information can be downloaded at: https://www.mdpi.com/article/10.3390/nu14194145/s1. Figure S1. Participant flowchart; Table S1. Baseline population characteristics of DO-HEALTH participants considered being robust at baseline; Table S2. Frequency distribution of frailty over the follow up of DO-HEALTH; Table S3. Interaction p-values of the exposure with the subgroup variables of interest.
