# Supplementary material for: Adherence to the Mediterranean Diet and Incidence of Pre-Frailty and Frailty in Community-Dwelling Adults 70+: The 3-Year DO-HEALTH Study
Source: Nutrients. 2022 Oct 6;14(19):4145. doi: 10.3390/nu14194145 (PMC9573135; doi:10.3390/nu14194145)
Supplement: Supplementary file 1 [file nutrients-14-04145-s001.zip › nutrients-1951456-supplementary.pdf]

SUPPLEMENTARY MATERIAL

Stephanie Gängler [1,2,†], Hanna Steiner [1,3,†], Michael Gagesch [1,2], Sophie Guyonnet [4,5], E. John Orav [6], Arnold von Eckardstein [7], Walter C. Willett [8,9] and Heike A. Bischoff-Ferrari [1,2,3,*]

[1] Center on Aging and Mobility, University Hospital Zurich, Zurich City Hospital-Waid and University of Zurich, 8006 Zurich, Switzerland
[2] Department of Aging Medicine and Aging Research, University Hospital Zurich and University of Zurich, 8006 Zurich, Switzerland
[3] University Clinic for Aging Medicine, Zurich City Hospital-Waid, 8037 Zurich, Switzerland
[4] Gérontopôle de Toulouse, Institut du Vieillissement, Centre Hospitalo-Universitaire de Toulouse, 31059 Toulouse, France
[5] UMR INSERM 1027, University of Toulouse III, Toulouse 31059, France
[6] Department of Biostatistics, Harvard T.H. Chan School of Public Health, Boston, MA 02115, USA
[7] Institute for Clinical Chemistry, University Hospital Zurich, 8091 Zurich, Switzerland
[8] Department of Nutrition, Harvard T.H. Chan School of Public Health, Boston, MA 02115, USA
[9] Department of Epidemiology, Harvard T.H. Chan School of Public Health, Boston, MA 02115, USA
* Correspondence: heike.bischoff@usz.ch
† These authors contributed equally to this work.

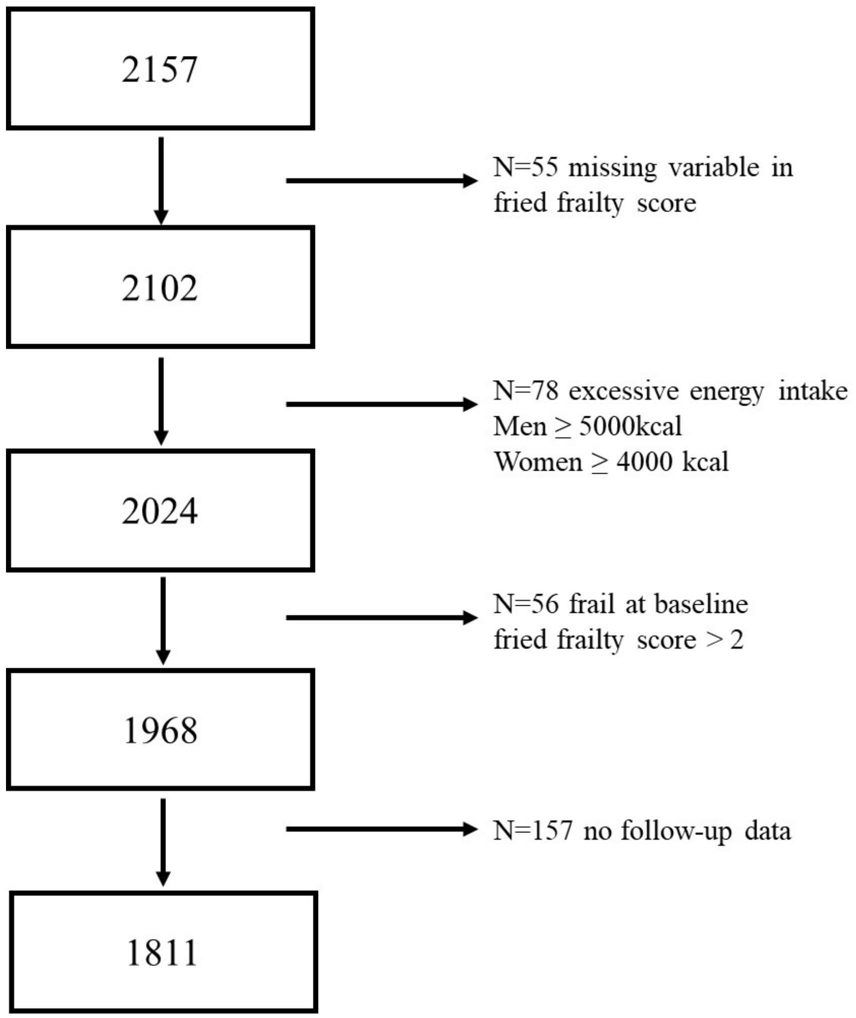

Figure S1. Participant flowchart

Table S1. Baseline population characteristics of DO-HEALTH participants considered being robust at baseline

Characteristics	Overall (n = 1035)	70-74 years (n = 630)	⩾75 years (n =405)	p-value
Age, mean (SD), years	74.41 (4.08)	71.78 (1.36)	78.49 (3.48)	<0.001
Women, No. (%)	575 (55.6)	353 (56.0)	222 (54.8)	0.749
Education, mean (SD), year	13.28 (3.87)	13.64 (3.75)	12.73 (4.01)	<0.001
BMI, mean (SD)[b]	26.08 (3.97)	26.10 (4.14)	26.05 (3.71)	0.841
MoCA score, median (IQR)[c],(0-30) points	27.00 (25.00, 28.00)	27.00 (25.00, 29.00)	27.00 (24.00, 28.00)	0.004
Sum of comorbidities, median (IQR)	1.00 (0.00, 2.00)	1.00 (0.00, 2.00)	2.00 (1.00, 2.00)	<0.001
Physical activity volume, median (IQR), MET-h/week[d]	30.00	31.45 (16.42, 57.62)	27.46 (12.81, 51.01)	0.010
Serum IL-6, median (IQR), ng/L	2.30 (1.50, 3.70)	2.10 (1.50, 3.30)	2.60 (1.80, 3.92)	<0.001
Serum CRP, median (IQR), mg/L	1.40 (0.80, 2.70)	1.40 (0.80, 2.50)	1.60 (0.80, 2.90)	0.027
Physical activity, N (%)				0.102
none	118 (11.4)	65 (10.3)	53 (13.1)	
1-2 times/week	327 (31.7)	190 (30.2)	137 (33.9)	
≥3 times/week	588 (56.9)	374 (59.5)	214 (53.0)	
Faller = Yes (%)	390 (37.7)	228 (36.2)	162 (40.0)	0.243
MedDietScore, mean (SD), (0-55) points	36.95 (4.85)	36.98 (4.72)	36.89 (5.06)	0.762
Energy intake, median (IQR), kcal/day	2428 (1950, 2927)	2411 (1932, 2915)	2434 (1958, 2947)	0.613

Abbreviations: IQR, interquartile range

[a] For these non-normal distributed variables, the Wilcoxon test was used.

[b] Body mass index (BMI) was calculated as weight in kilograms divided by height in meters squared. Higher BMI values reflect overweight (⩾25) and obesity (⩾30).

[c] The Montreal Cognitive Assessment (MoCA) is a screening test for mild cognitive dysfunction and has a range of 0 to 30 points, in which higher scores are better and scores greater than 26 suggest normal cognitive function.

[d] Weekly volume of physical activity was estimated based on the Nurses' Health Study questionnaire on physical activity, in which energy expenditure of different activities in metabolic equivalent tasks (METs) of activities based on the Compendium of Physical Activities were summed over the previous week.

Table S2. Frequency distribution of frailty over the follow up of DO-HEALTH

	Year 1	Year 2	Year 3
**Robust, n (%)**	839 (48.4)	704 (41.8)	657 (39.8)
**Pre-frail (1-2 points), n (%)**	853 (49.2)	918 (54.5)	931 (56.4)
**Frail (⩾3 points), n (%)**	43 (2.5)	62 (3.7)	63 (3.8)

Table S3. Interaction p-values of the exposure with the subgroup variables of interest.

	Baseline MedDietScore		Change in MedDietScore from baseline ($\Delta_{Y3\text{-}BL}$)	
	**frailty**	**pre-frailty**	**frailty**	**pre-frailty**
**Sex**	0.7515	0.0826	0.9261	0.9577
**Age >75 years**	0.4124	0.4241	0.5889	0.5210
**Physical activity level (active vs inactive)**	0.6017	0.9625	0.4691	0.2534
**BMI > 30**	0.8114	0.6358	0.6826	0.1343
**Study site**	0.3643	0.2718	0.0514	0.2028
